# Low-cost programable stroboscopic illumination with sub-microsecond pulses for high-throughput microfluidic applications

**DOI:** 10.1016/j.ohx.2022.e00367

**Published:** 2022-10-04

**Authors:** Marko Tuljak, David Lajevec, Rok Štanc, Špela Zemljič Jokhadar, Jure Derganc

**Affiliations:** a Institute of Biophysics, Faculty of Medicine, University of Ljubljana, Vrazov trg 2, 1000 Ljubljana, Slovenia; b Chair of Microprocess Engineering and Technology—COMPETE, University of Ljubljana, Večna Pot 113, 1000 Ljubljana, Slovenia

**Keywords:** Deformability cytometry, Velocity measurement, Microfluidics, Stroboscope, Teensy, Arduino

## Abstract

To visualize fast-moving objects in microfluidic applications, the image acquisition time must be on the order of a microsecond or less. Commercial imaging systems capable of such short exposure times may be too expensive for many research laboratories. We have therefore developed a low-cost stroboscopic illumination for transmitted-light microscopy based on a high-power LED that can be coupled to a standard industrial camera and provides exposure times on the order of 500 ns. The system is designed to be easily mounted on a standard condenser of an inverted microscope. The illumination is controlled by a fast Arduino-compatible Teensy® 4.0 development board, and the illumination parameters can be set from a PC via a graphical user interface written in Python. The system has been successfully used for high-throughput cell phenotyping using deformability cytometry on a Nikon TE2000 microscope, as well as for droplet microfluidic on an old Olympus inverted microscope.

## Graphical abstract

**Figure:**
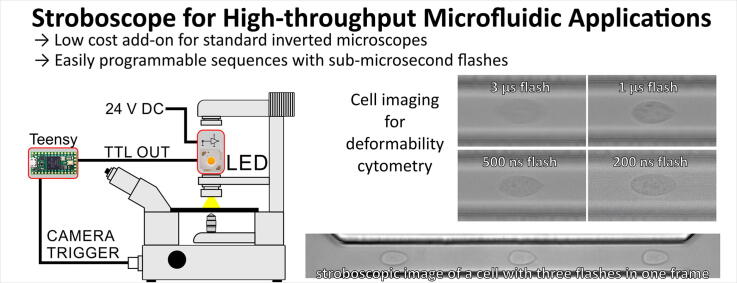

## Specifications table

**Table:** 

Hardware name	*Programable Stroboscopic LED Illumination for Microscopy*
Subject area	General
Hardware type	Imaging tools
Closest commercial analog	*AcCellerator/DeCellerator by Zellmechanik Dresden GmbH*
Open source license	*GNU General Public License (GPL) 3.0*
Cost of hardware	*120 EUR*
Source file repository	*https://osf.io/fnhm7/*

## Hardware in context

In typical high-throughput microfluidic applications, such as microdroplet analysis [1], cell imaging flow cytometry [2], or deformability cytometry [3], objects move at velocities on the order of 1 m/s. To put this velocity in context: If scaled, a cell with a diameter of 20 µm moving at 1 m/s is comparable to a 20 cm soccer ball moving at 10 km/s. Objects moving at such high speeds cannot be visualized with standard cameras that have acquisition times on the order of 10 µs. To avoid blurring, exposure times must be in the microsecond range or less. For many laboratories, commercial high-speed cameras capable of such short exposure times are prohibitively expensive. An alternative approach to acquire sharp images of fast-moving objects is the stroboscopic technique, i.e., to use a standard camera and a short bright flash to illuminate the object [1], [2], [3]. While standard microscope illumination is too slow for stroboscopic illumination, microscopy systems based on high-power light emitting diodes (LEDs), such as Zellmechanik’s DeCellerator, can provide fast acquisitions, but they still cost tens of thousands of dollars.

Over the past decade, low-cost open-source illumination systems based on high-power LEDs have been successfully applied to a variety of microscopy techniques that require illumination with short flashes, from particle image velocimetry [4], [5] to fast multi-color fluorescence and optogenetic applications [6], [7], [8]. In addition, LEDs in pulsed mode have been shown to operate at voltages that are much higher than their nominal voltage for continuous operation [4], which makes them particularly suitable for stroboscopic applications. To control the LED flashes, systems typically employ Arduino-compatible development boards [6], [7], [8], [9], which can output easily programable pulses of varying lengths. However, accurate timing of the pulses in the sub-microsecond range is limited by the relatively low speed of Arduino.

Inspired by open-source work on high-power LEDs in microscopy, we have developed a simple stroboscopic LED illumination for transmitted light microscopy that can deliver programmable sub-microsecond pulses and is suitable for imaging in high-throughput microfluidic applications. We used an Arduino-compatible Teensy® 4.0 development board which runs at 600 MHz (the standard Arduino runs at 16 MHz) and can output pulses in the range of 100 ns. The system is designed to be easily mounted on a standard inverted microscope without requiring changes to existing light paths or imaging modalities, which is critical in laboratories where one microscope is shared by multiple experimental applications. The Teensy code for pulse timing is only about 20 lines long, making it easy to customize for non-conventional pulse sequences. In addition, all the parameters of the stroboscopic illumination can be controlled via a simple graphical user interface (GUI) written in Python, allowing the system to be used by novice users without knowledge of Arduino programing.

## Hardware description

**Fig. 1:**
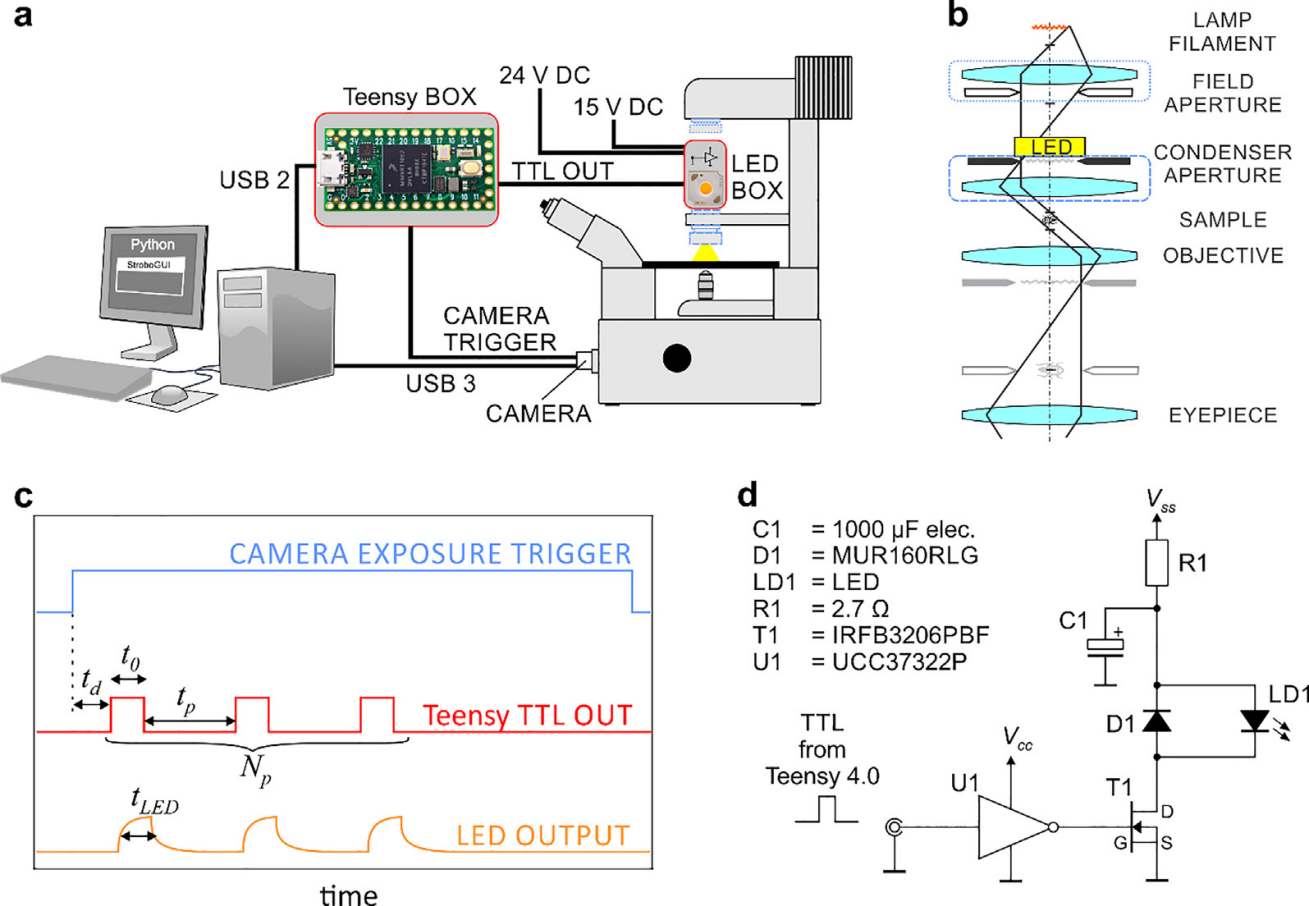
Description of the stroboscopic LED illumination setup for transmitted light microscopy. a) Experimental setup. The stroboscopic LED illumination consists of two boxes (marked in red) connected to the microscope camera and the PC. The LED is positioned on the microscope condenser. The condenser is marked with blue dashed lines, and the collector with blue dotted lines. b) Schematic of the LED position in the optical path of transmitted light illumination. The LED is marked in yellow and the optical images of the elements of the standard transmitted illumination are gray. The condenser and collector units are marked with blue dashed and dotted lines, respectively. The LED is positioned behind the condenser aperture near the back focal plane of the condenser, i.e., at the position of the optical image of the filament in the standard illumination light path, which ensures uniform illumination of the sample. c) A schematic representation of the illumination parameters. d) A schema of the LED driver circuit. (For interpretation of the references to color in this figure legend, the reader is referred to the web version of this article.)

The experimental setup is schematically shown in Fig. 1A. The stroboscopic LED illumination consists of two modules: a 3D printed box for the LED and its driver, mounted on the microscope condenser, and a control box comprising a Teensy 4.0 development board. The LED is positioned approximately in the back focal plane of the condenser (Fig. 1B), which ensures uniform illumination of the sample. The principle of operation is as follows (Fig. 1C): When the camera starts image acquisition, it outputs a TTL trigger signal that is detected by the Teensy. The Teensy then sends $N_{p}$ TTL pulses with width $t_{0}$ to the LED driver. In this way, one camera frame is illuminated by $N_{p}$ flashes, creating the stroboscopic effect. The inter-pulse width is $t_{p}$, and the delay between the camera exposure and the first pulse is $t_{d}$. The LED emits light for the duration of the TTL pulses from the Teensy, but its response is not instantaneous and therefore the actual LED flashes exhibit an exponential rise and fall. The width of the LED pulses at half-maximum (FWHM) is referred to as $t_{\mathrm{LED}}$. The flashing parameters on the Teensy can be set via a USB connection from a PC running a graphical user interface (GUI) written in Python. The schematic of the LED driver is shown in Fig. 1D [4], [10]. The camera is connected to the PC via USB 3, which allows image acquisition at 160 full frames per second and up to 1000 frames per second if the region of interest is reduced.

• Timing of pulses with Teensy 4.0 is software-based. It relies on the build-in function delayNanoseconds(), which is not a standard Arduino function and is only available on Teensy 4.0. With the presented system, the durations of the shortest Teensy TTL OUT pulse width and the shortest interpulse width are approximately $t_{0}$ = 50 ns, $t_{p}$ = 50 ns.
• The duration of the shortest usable LED flash is approximately $t_{\mathrm{LED}}$ = 200 ns. It is limited by the rise/fall time of the LED and its driver, not by the speed of the Teensy. The final intensity on the camera depends on the LED pulse width ($t_{\mathrm{LED}}$), the LED driving voltage ($V_{\mathrm{SS}}$), the brightness of the objective, and the microscope’s port (e.g., 80/20 or 100).
• The enclosure for the LED illumination is 3D-printed and fits into a standard condenser unit of Nikon inverted research microscopes (e.g., Eclipse-2000, Ti, Ti-2). It can be mounted and unmounted in seconds and does not interfere with other imaging techniques used on the microscope. The LED is positioned near the back focal plane of the condenser and provides uniform illumination in the plane of the sample. The 3D-printed housing is optional – one can also use a handmade cardboard/duct tape housing.
• The illumination parameters can be controlled via a GUI on a PC, so that the system can be used by an unexperienced user.
• Software-based timing of pulses allows easy tinkering with pulse sequences and does not require in-depth knowledge of the hardware.

## Design files summary

**Table:** 

Design file name	File type	Open source license	Location of the file
3D-printed LED housing	STL	GNU General Public License v3.0	https://osf.io/yjwvr
3D-printed LED holder	STL	GNU General Public License v3.0	https://osf.io/z5p8r
3D-printed lid for LED housing	STL	GNU General Public License v3.0	https://osf.io/et4ax
StroboTeensy.ino	Arduino IDE	GNU General Public License v3.0	https://osf.io/mkras
StroboGUI.py	Python	GNU General Public License v3.0	https://osf.io/9fs8a

The design files comprise three STL files for 3D printing of the LED illumination housing, a script for the Teensy development board, and a Python GUI used to control the system from a PC. The LED housing described in this paper is designed to fit a standard condenser of a Nikon inverted research microscope.

## Bill of materials summary

**Table:** 

Designator	Component	Number	Cost per unit	Total cost	Source of materials	Material type
	Teensy 4.0	1	21.66 €	21.66 €	https://www.exp-tech.de	Semi-conductor
	Teensy Header Kit	1	1.56 €	1.56 €	https://www.exp-tech.de	
U1	MOSFET Driver UCC37322P	1	2.11 €	2.11 €	farnell.com 8,463,034	Semi-conductor
T1	Power MOSFET IRFB3206PBF N Channel, 60 V	1	2.79 €	2.79 €	farnell.com 1,436,949	Semi-conductor
LD1	LED CMA1303-0000-000C0H0A30G WARM WHITE, 9 V, 780LM	1	2.26 €	2.26 €	farnell.com 3,227,245	Semi-conductor
D1	Fast diode MUR160RLG 600 V, 1 A	1	0.40 €	0.40 €	farnell.com 9,557,008	Semi-conductor
C1	Electrolytic Capacitor 1000 µF, 50 V	1	0.50 €	0.50 €	soselectronic.de 64,389	Aluminum
R1	Resistor, 2.7 O, CFR, 500 mW, ±5 %, 350 V	1	0.20 €	0.20 €	farnell.com CFR50J2R7	Metal
J1	Coaxial BNC Connector	3	4.66 €	13.98 €	farnell.com 1-1337452-0	Metal/plastic
J2	DC Power Connector (COB LED)	1	1.56 €	1.56 €	farnell.com 1614 10	Metal /plastic
J3	DC Power Connector (MOSFET Driver)	1	1.14 €	1.14 €	farnell.com 27-3157	Metal
	DIP Socket	1	0.30 €	0.30 €	farnell.com 2227MC-08-03-18-F1	Metal/plastic
	Wires	2 m	0.60 €	1.20 €	https://www.tasker.it C131-1x0.35 mm^2^	Copper
	Coaxial cable with BNC connectors, 1 m	1	7.53 €	7.53 €	farnell.com MC002871	
	Power Supply	2	23.57 €	47.14 €	farnell.com MW7H380GTGS	
	Plastic Enclosure, Junction Box	1	2.21 €	2.21 €	farnell.com ENN05002	Plastic
	Micro USB B to USB A Male to Male cable	1	€ 2.09	2.09 €	farnell.com PSG90890	
	Heatsink 1 cm × 1 cm × 1 cm	1	1.93 €	1.93 €	farnell.com ICK BGA 10 X 10 X 10	Aluminum
	Adhesive Heat Sink Pad, Graphite	1	2.90 €	2.90 €	farnell.com ILA-TIM-LES13-2A	
	1″ square protoboard (Sparkfun)	1	1.87 €	1.87 €	eu.mauser.com PRT-08808	
	50 mm M4 screw with a bolt and 2 washers	1	1 €	1 €	Local hardware store	
	(2.2x4.5) mm self-tapping screw with a washer	3	1 €	1 €	Local hardware store	

The LED illumination was tested with a Nikon Eclipse TE2000 inverted research microscope with a 40x objective and a Ximea industrial camera with Sony IMX-174 chip (MC023MG-SY-UB, the cost of a new camera is about 1000 €) on an 80/20 microscope front port. Note that a camera with “global shutter” is required for stroboscopic illumination. Also, the camera must have an I/O port for triggering external devices (the I/O cable is usually provided by the camera manufacturer at additional cost). The housing for the LED illumination was printed with a Prusa i3 MK3 3D printer. It consumed approximately 100 g of the standard PLA filament (the cost of the filament was less than 5 €).

## Build instructions

### LED driver

The LED driver design follows the one described in detail in [4], [10]. The whole LED driver electronic circuit (Fig. 1D) fits a small 1-inch square protoboard (Supplementary Fig. 1). Electronic components on the protoboard are assembled using wires from a Jumper Wire Kit (e.g., MC001810, https://www.farnell.com, 6.67 €) and soldered on its back side.

A small 1x1x1 cm heatsink is mounted on the back side of the LED by a self-adhesive thermal pad (Supplementary Fig. 1). The LED is then connected to the LED driver by two 10 cm wires. It is advisable to keep the connections between the components as short as possible to avoid noise and the effect of parasitic inductance introduced by the wires [4], [10]. Because the current for the LED flashes is provided by a capacitor C1, a standard 24 V DC power supply is enough for delivering $V_{\mathrm{SS}}$.

### Teensy box

**Fig. 2:**
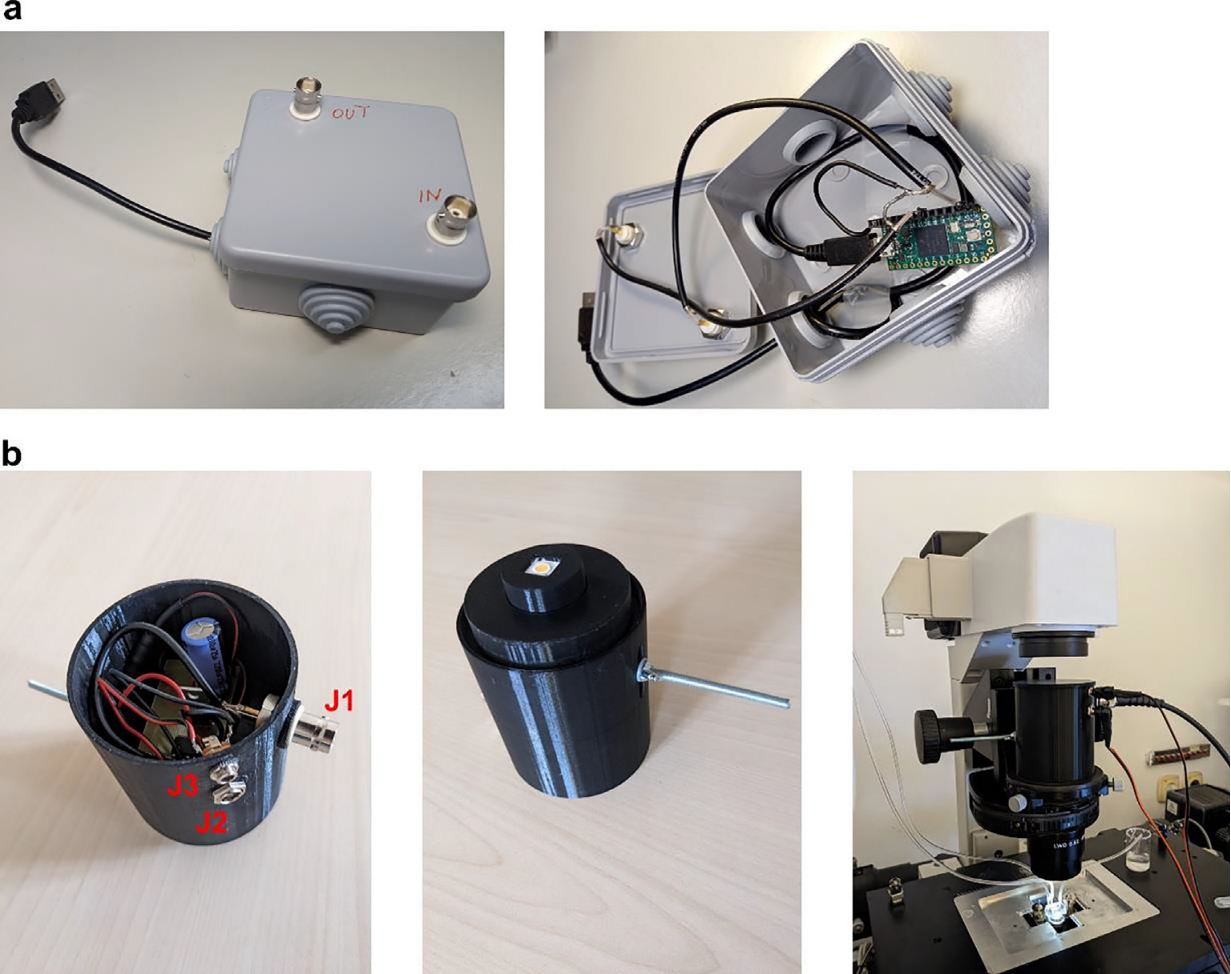
The housings for the Teensy development board (a) and the LED and its driver (b). The image on the right shows the assembled system mounted on a condenser of a Nikon TE2000 inverted microscope during a microfluidic application.

For easier soldering of connectors to the Teensy board we recommend using the Teensy Header Kit, which is sold separately. To protect the Teensy board, it is secured in an off-the-shelf plastic enclosure (Junction Box) that has openings for the Micro USB B to USB A Male to Male cable, which connects the Teensy to a PC, and two coaxial connectors (J1) for the camera trigger TTL IN and the Teensy TTL OUT (Fig. 2a, Supplementary Fig. 2). The power for the Teensy is provided via USB. TTL IN is connected to PIN 14 on the Teensy, and TTL OUT to PIN 19 (any other suitable combination of pins can be used as well).

Teensy pins accept only 3.3 V TTL signals. If the camera outputs a 5 V TTL, the signal has to be lowered to 3.3 V before reaching the Teensy, using for example a logic level converter, such as SparkFun Logic Level Converter (PRT-14765).

### The LED box for the LED and its driver

The 3D-printed LED box consists of three separate parts (Fig. 2b, and Supplementary Fig. 3):

1. The main cylindrical housing that fits into the microscope’s condenser unit.
2. The narrow cylindrical holder for the LED, which fits into the housing and slides vertically to fine tune the position of the LED with respect to the condenser lens.
3. The lid for the housing.

The housing has side-openings for three connectors: a coaxial connector for the TTL trigger from Teensy (J1), a DC power connector (J3) for $V_{\mathrm{CC}}$ (15 V), which powers the MOSFET driver U1, and a DC power connector (J2) for $V_{\mathrm{SS}}$ (24 V), which charges the capacitor C1 powering the LED. The 15 V and 24 V power connectors are different so that the user cannot accidentally connect wrong voltages. Also, separate connectors allow flexibility to adjust Vss without affecting Vcc (if needed). Make sure that the power supplies are set to correct connectors, voltages and polarities (see the power supply manual). The housing is closed by the lid, which has only an esthetic and no functional role.

The LED is mounted into the narrow cylindrical holder by two self-tapping screws with washers fastened directly into the 3D-printed PLA. The cylindrical holder is then inserted into the main housing and secured by a M4 screw and bolt. The protoboard is secured on the housing with a single self-tapping screw and a washer.

The STL files for 3D printing provided in this paper are for a LED housing that fits on a standard condenser for Nikon inverted research microscopes. For use with a different microscope, the dimensions of the housing need to be adjusted accordingly. Note that the 3D-printed housing for the LED is optional – the illumination works just as well with an improvised housing made of duct tape and cardboard (Supplementary Fig. 4).

### Installing the software

The software for the stroboscopic illumination consists of two parts: the StroboTeensy script for the Teensy and the StroboGUI Python script for the graphical user interface (GUI) on the PC. Once the StroboTeensy is installed on the Teensy, all the illumination parameters can be set via the StroboGUI.

1. Download and install Arduino environment (Arduino IDE) on the computer (www.arduino.org). Note that only certain versions of Arduino IDE are supported by Teensy, see https://www.pjrc.com/teensy/td_download.html.
2. Download and run the Teensy driver, i.e., the Teensyduino installer (https://www.pjrc.com/teensy/td_download.html). After Teensyduino is installed, the Teensy can be used as any other Arduino.
3. Download the StroboTeensy script from the repository and install it on the Teensy (follow the instructions provided by Arduino IDE, https://www.arduino.cc/en/software).
4. Download the StroboGUI Python script from the repository and run it as any other Python script. The script requires “pySerial” module, which is a standard module in many Python distributions (for installation instructions refer to your Python environment documentation). For easier operation it is advised that you make an executable program which can than run directly from a PC without using Python environment.

## Operation instructions

The Teensy is connected to the PC via USB, the Teensy TTL IN to the “I/O out” port on the camera via the I/O cable provided by the camera manufacturer, and the Teensy TTL OUT to the LED driver via a coaxial cable. The 24 V and 15 V power supplies are connected to the appropriate connectors on the LED housing. The LED housing is mounted onto the microscope condenser.

Once the StroboTeensy script is installed on the Teensy and the Teensy is plugged into a PC USB port, it will start listening for the trigger signal from the camera and generating pulses with the flashing parameters that are saved in its memory (the initial set of parameters is defined when the StroboTeensy script is installed on the Teensy). The parameters remain saved in the Teensy even after it is powered off.

To change the flashing parameters, one can change their values in the StroboTeensy script and re-install the script via Arduino IDE. An alternative method is to use the StroboGUI script on the PC, which does not require Arduino IDE. When the StroboGUI starts, the user must first specify the PC COM port connected to Teensy. The COM port of the Teensy can be determined from the Adruino IDE/Tools/Ports or from the Device Manager/Ports (COM&LPT), and usually doesn’t change unless additional USB devices are plugged into the PC.

Image acquisition is performed using the software provided by the camera manufacturer. Note that the camera must be set to output the exposure trigger signal, i.e., the camera I/O must be “on” for the entire duration of exposure (most cameras do not output this signal by default). It is advisable that, before the first use, the triggering is verified by an oscilloscope – for short acquisition times, the TTL signal from the camera may not reach the threshold required by Teensy (Supplementary Fig. 5).

If the LED is not centered in the optical axis of the transmitted light path, it can be centered with the knobs for centering the condenser aperture. Non-ideal centering shows as an asymmetrical halo around objects in the image (Supplementary Fig. 6). Vertically, the LED can be adjusted by moving the condenser unit or by using the screw on the side of the LED housing. If the condenser aperture is fully open, the screw allows to position the LED positioned exactly in the back focal plane of the condenser. However, we have noticed that the precise vertical position of the LED does not visibly affect the image quality in our application, therefore we simply adjust the vertical position of the condenser so that the intensity on the camera is maximal.

Potential safety hazards: At full power, the flashes from the LED are very bright – avoid direct eye contact.

## Validation and characterization

**Fig. 3:**
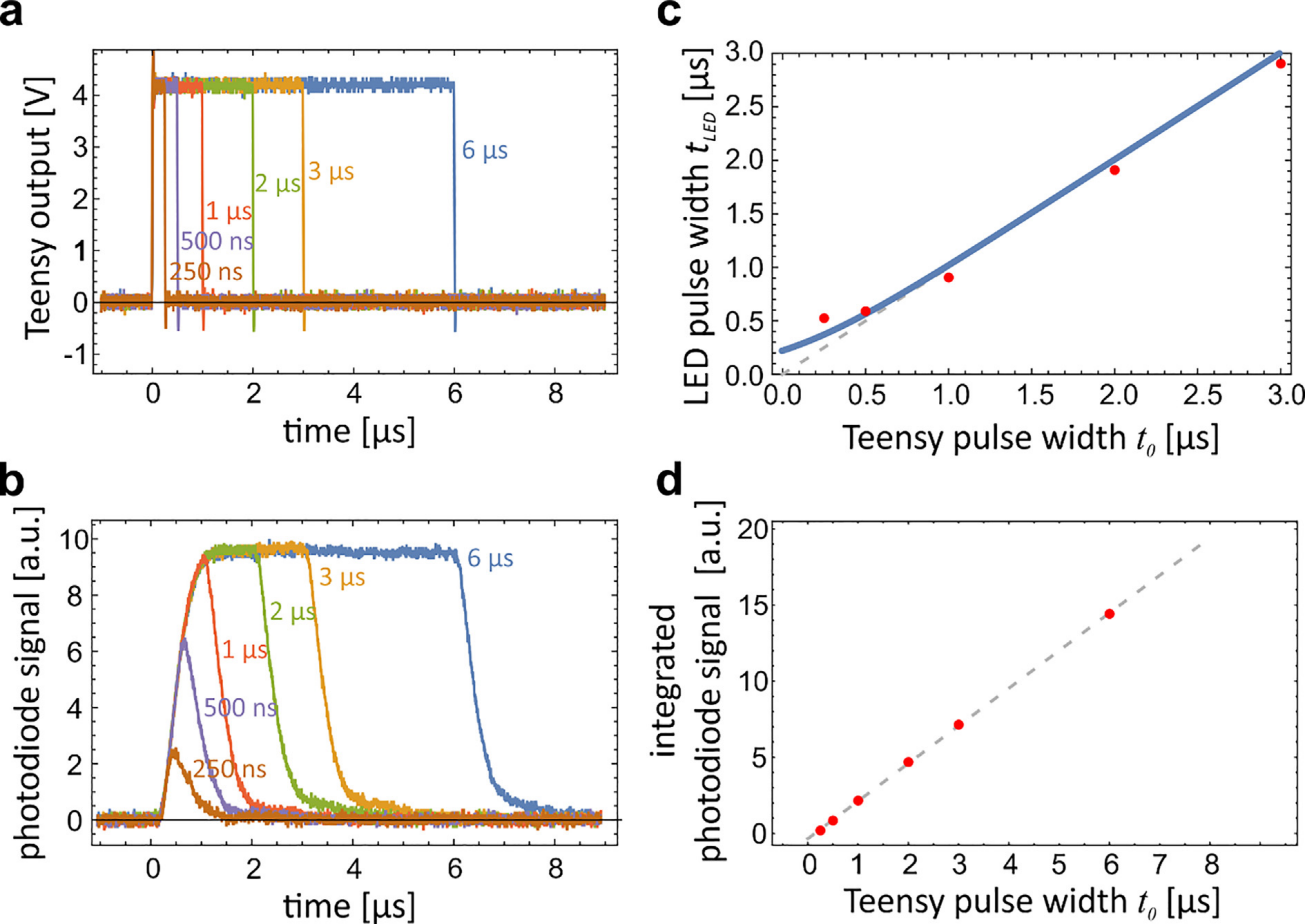
Characteristics of LED flashes measured with a photodiode and an oscilloscope. a) The shape of Teensy output TTL pulses at different values of Teensy pulse width $t_{0}$. b) LED flashes as measured with a photodiode at different values of $t_{0}$. In all cases, the characteristic time of the exponential rise of the signal is approximately τ = 300 ns. c) The LED pulse width $t_{\mathrm{LED}}$ as a function of $t_{0}$. The red dots denote the measurements, the blue line is the theoretical curve obtained from Eq. (1) and τ = 300 ns, and the gray dashed line denotes values $t_{\mathrm{LED}}=t_{0}$. d) The numerically integrated signal detected by the photodiode as a function of $t_{0}$. The gray dashed line denotes the best linear fit. (For interpretation of the references to color in this figure legend, the reader is referred to the web version of this article.)

First, we evaluated the TTL pulses generated by the Teensy 4.0 (Fig. 3a). The oscilloscope (Tektronix TDS 1001B, 40 MHz) showed that the shortest Teensy pulse duration using the StroboTeensy script was approximately $t_{0}$ = 50 ns (Supplementary Fig. 7). Note that because the pulse triggering is software-based, it is inherently subjected to delays induced by Teensy code execution. The StroboTeensy script compensates for these delays with a variable TeensyDelayNano. In this way, if the user sets the Teensy pulse width to 100 ns, the Teensy TTL pulse width is indeed 100 ns.

While Teensy can output short square TTL pulses, the LED flashes exhibit an exponential rise and fall with characteristic time that depends on the set-up, e.g., the speed of the LED and its driver, and the length of the wires [4]. Measuring the LED intensity with a photodiode (Hamamatsu S5973) revealed an approximately exponential rise of the signal with a characteristic time τ = 300 ns (Fig. 3b). Note that this characteristic time may not depend only on the LED response but also on the response of the photodiode detection system, however, with our instrumentation we were not able to distinguish between these two effects. The shortest detectable flash was reached at approximately $t_{0}$ = 200 ns. For Teensy pulses shorter than approximately 1.5 µs, the LED intensity started to decrease before reaching the maximum value.

To theoretically model the LED flash width $t_{\mathrm{LED}}$, we assumed that the flash intensity exponentially rises and falls with a characteristic time τ (Supplementary Fig. 8). By defining the LED flash width $t_{\mathrm{LED}}$ as the full width at the pulse half-maximum (FWHM), analytical integration yields that the LED flash width is equal to.

(1) $${t_{\mathrm{LED}}=t}_{0}+\tau \ln\left(1+e^{-\frac{t_{0}}{\tau }}\right)$$

Correspondingly, the integrated LED intensity, which is proportional to the number of emitted photons, is.

(2) $$\int_{0}^{\infty }I\left(t\right)dt{=I_{0}t}_{0},$$

where I(t) is the instantaneous LED intensity and $I_{0}$ its saturating value.

Fig. 3c shows the theoretical and the measured LED flash width $t_{\mathrm{LED}}$ as a function of the Teensy pulse width $t_{0}$. The measured data were in qualitative agreement with the theoretical prediction. For long pulses, the flash width was linear with Teensy pulse width and approximately 100 ns shorter. For short pulses, the measured flash width approached the limit of about 500 ns, regardless of the Teensy pulse width (according to theoretical model, the limiting value is $\lim_{t_{0}\to 0}t_{\mathrm{LED}}=\tau \ln2)$. In agreement with theoretical prediction, the numerically integrated photodiode signal was proportional to the Teensy pulse width for all pulse widths (Fig. 3d). Thus, if the system is used in applications that do not require extremely short exposure times, the brightness of the illumination can be simply adjusted by changing the Teensy pulse width. For applications that require the shortest flash possible, the brightness can still be adjusted by the LED driving voltage $V_{\mathrm{SS}}$ (see below).

**Fig. 4:**
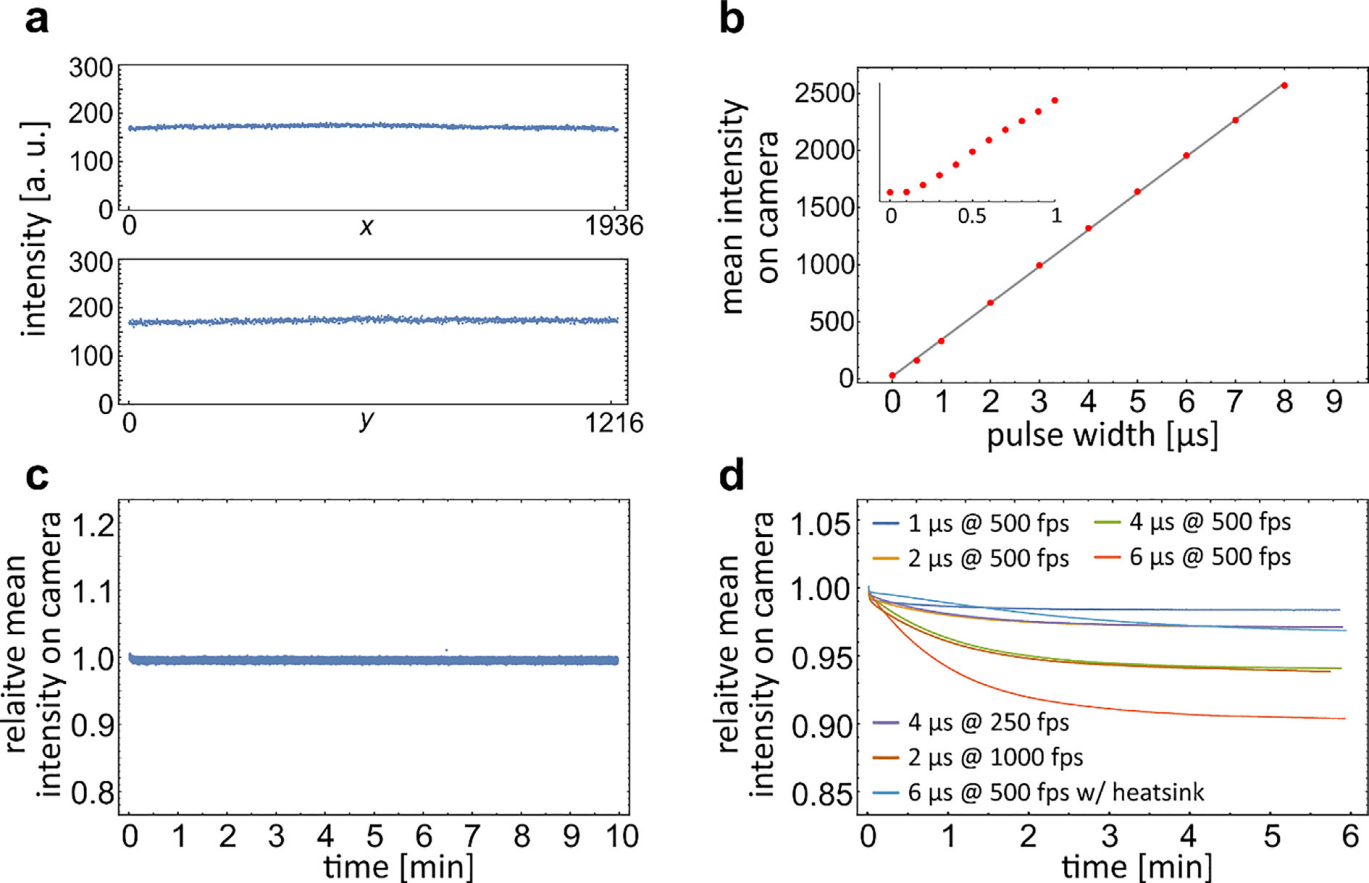
Validation of the illumination system with a microscope. a) Horizontal and vertical intensity profiles acquired by the camera in the sample plane. b) Mean intensity on camera, captured in the sample plane, as a function of Teensy pulse width $t_{0}$. The measurements in the sub-microsecond range are enlarged in the small inset panel. c) Stability test. Relative mean intensity on camera for 300,000 fames acquired during 10 min with 250 ns flashes at 500 frames per second. Only one frame (at approximately 6.5 min) showed intensity that deviated noticeably from the average. d) Thermal test. Relative mean intensity on camera as a function of time for non-cooled LED and duty cycles from 0.0005 (1 µs at 500 fps) to 0.003 (6 µs at 500 fps). Thermal characteristics were significantly improved after mounting a small passive heatsink to the backside of the LED (light blue line). (For interpretation of the references to color in this figure legend, the reader is referred to the web version of this article.)

Finally, we analyzed the LED illumination with the microscope. Since the LED illumination bypasses the collector of the standard Koehler illumination, we first checked the flatness of the illuminated field. We found that the position of the LED near the back focal plane of the condenser provided an evenly illuminated field of view (Fig. 4a), comparable to that obtained with the full Koehler illumination comprising the field aperture. We then measured how the intensity of the acquired image depended on the Teensy pulse width $t_{0}$ (Fig. 4b). In agreement with the photodiode measurements and the theoretical model (Fig. 3d), we found that the intensity was linear with $t_{0}$ down to 200 ns, where it reached the thermal noise of the camera.

To evaluate the stability of the LED flashes duration, we measured the variation of the intensity on camera for long acquisition runs. Fig. 4c shows a typical 10-minute run with 250 ns pulses acquired at 500 fps. Among the 300,000 acquired frames the intensity of only one frame deviated from the mean value for more than 0.5 %. The standard deviation of the intensities for the entire run was on the order of 0.1 %, which was comparable to the standard deviation of intensities obtained at a similar absolute intensity level by the standard halogen illumination of the microscope. Note that the intensity stability is markedly worse if the Teensy code is not optimized for accurate timing (Supplemental Fig. 9).

To assess the thermal properties of the set-up, we analyzed long acquisition runs at increasing LED duty cycles. An increased temperature reduces the LED luminosity, therefore it shows as a decrease in the intensity acquired by the camera. In addition, we used a thermocouple to measure the temperature at the back side of the LED. Fig. 4d shows how the LED luminosity decreased during acquisitions at different duty cycles. For a non-cooled LED and a typical duty cycle of 0.0005, i.e., 1 µs flashes at 500 fps, the intensity decreased for approximately 2 %, and the temperature stabilized at 28 °C (the room temperature was 22 °C). As expected, the thermal response depended only on the integral LED duty cycle but not on the distribution of the flashes, e.g., 2 µs flashes at 1000 fps resulted in the same temperature increase as 4 µs flashes at 500 fps. If the duty cycle was increased to 0.003, the luminosity fell for approximately 10 % and the temperature reached 59 °C, which is close to the thermal limit for the PLA filament used for the 3D printed housing. The thermal properties significantly improved after a small passive heatsink was mounted on the back side of the LED.

**Fig. 5:**
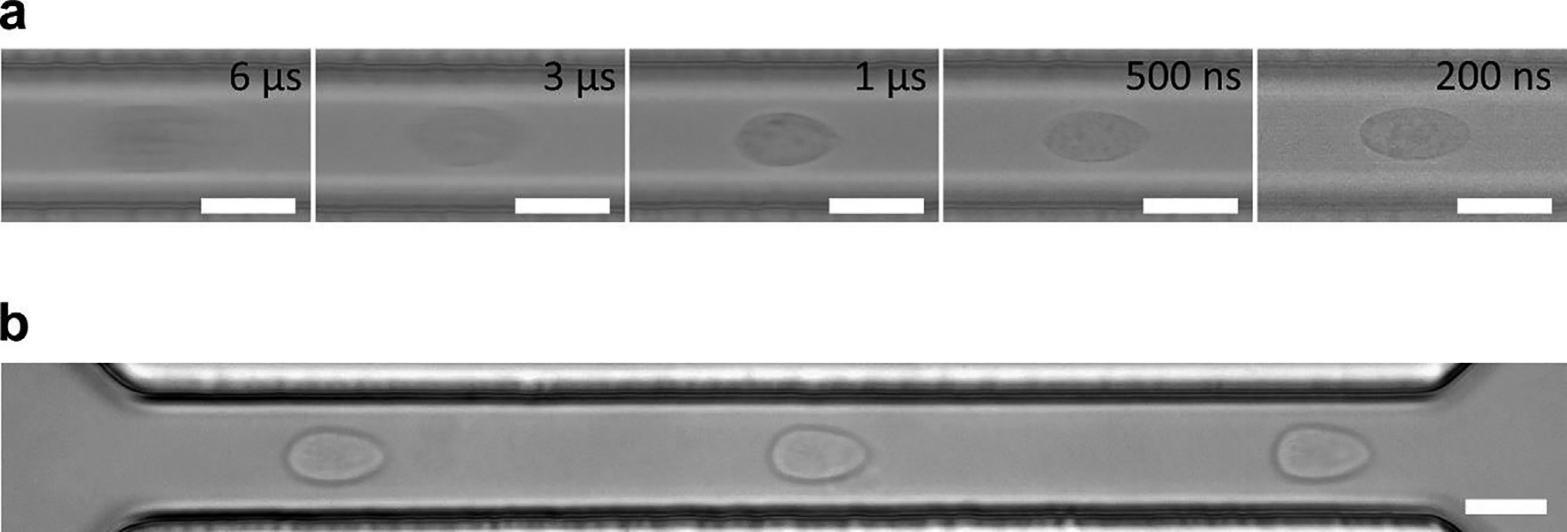
Images of cells in deformability cytometry essay, captured with a decreasing Teensy pulse width $t_{0}$. The camera gain was adjusted for each acquisition. The microchannel was 30 µm wide and the flow velocity was approximately 2 m/s. d) A stroboscopic image of a cell in a microchannel, acquired with three consecutive 1 µs flashes delayed 60 µs apart ($t_{0}$ = 1 µs, $t_{p}$ = 60 µs, $N_{p}$ = 3). The microchannel width and the flow velocity were the same as in c). Scale bars: 20 µm.

To assess the LED stroboscopic illumination in a high-throughput microfluidic application, we tested the system with deformability cytometry of Jurkat cells. Except for the imaging part, the experimental setup has been described previously [11]. In short, the cells are pushed one-by-one at high speed through a narrow microfluidic channel where they deform due to high shear forces [3]. The individual cells are imaged, and their deformation is assessed from the image. The need for short acquisition times in this application is evident from Fig. 5a, where cell images obtained at various Teensy pulse widths are shown. With 6 µs pulses, the cells are barely visible, but their images become sharp with flashes of 1 µs and less. With the setup presented, we were able to analyze 20,000 cells in 5 min.

The flashing parameters of the presented LED stroboscopic illumination can be easily adjusted to specific needs. Fig. 5b shows a cell that was illuminated with three successive flashes, so that its passage through the entire length of the microchannel can be followed. In addition, due to accurate timing between flashes, the system can be used for straightforward measurements of cell velocity, $v=\Delta x/(t_{0}+t_{p})$, where Δx is the distance traveled by the cell between two successive flashes. Accordingly, the system can be as well used for particle image velocimetry [4], [5].

**Fig. 6:**
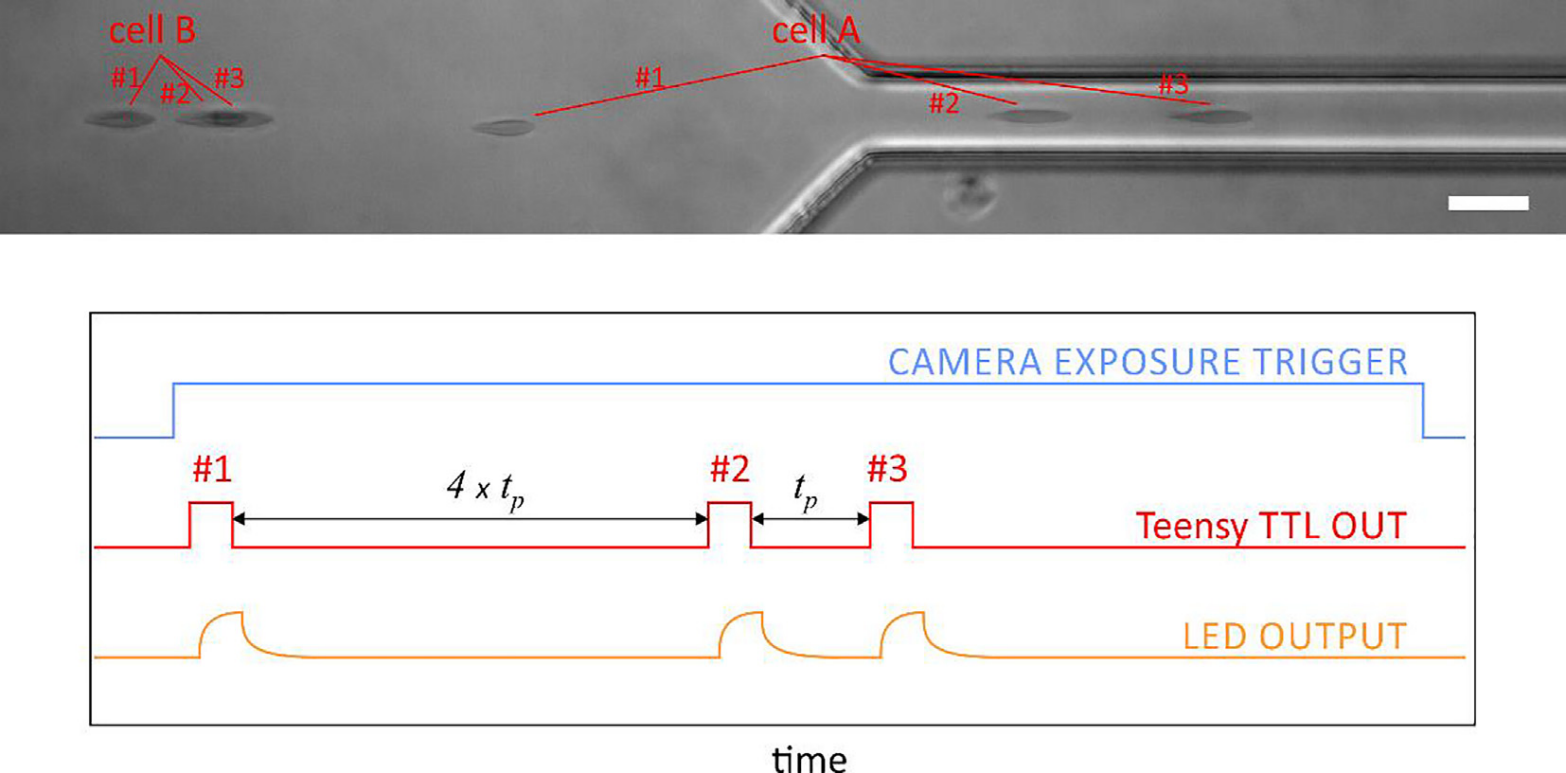
An example of cell deformability image acquired with a modified pulse sequence (schematically shown below the image). In this example, the Teensy code was modified to generate flashes with varying inter-pulse widths. In this way, one can follow a cell in flow that accelerates significantly as the cell enters a narrow microchannel. The image shows two red blood cells as they approach the channel narrowing. The inter-pulse duration between flashes #1 and #2 was four time longer than the one between flashes #2 and #3. Cell A started in a slow flow and then accelerated in the narrow channel. Cell B remained in the slow flow during all three flashes, so its last two images overlapped. Scale bar: 20 µm. (For interpretation of the references to color in this figure legend, the reader is referred to the web version of this article.)
• The LED in the presented set-up is driven by 24 V, which is supplied by an off-the-shelf power supply and does not pose a safety risk. However, it has been shown that LEDs are usually damaged by thermal effects rather than voltage, and that in the stroboscopic mode they can withstand voltages much higher than the nominal voltage and thus produce much brighter flashes than in continuous mode [4], [10]. The LED used in our system was tested up to $V_{\mathrm{SS}}$ = 59 V (the highest DC available in our lab, Supplementary Fig. 10). Note that, if higher voltages are used, C1 must be replaced by a capacitor that can tolerate higher voltages.
• Good cooling is essential for system operation at high duty cycles. A simple passive heat sink is adequate for duty cycles up to about 0.001, but active cooling is recommended for higher duty cycles [4], [10].
• For applications with higher duty cycles or higher trigger frequencies, the values of C1 and R1 may need adjustment, e.g., by decreasing R1 to shorten the characteristic time for charging C1 (if C1 is not fully charged between successive flashes, the intensity of the LED would not reach its maximal value). Also, a more powerful power supply might be required [4], [10].
• The experimental set-up presented here is modular so that the Teensy and the LED driver can be used separately in other applications. If needed, the two can also be housed in the same enclosure. Also, long wires are used to connect the LED to the driver, so that the vertical position of the LED can be adjusted independently from position of the LED driver in the main housing. For applications that require very short pulses, a more compact solution would be advantageous, because shorter connecting wires would improve the characteristics of the illumination unit [4], [10].

The stroboscopic illumination presented in this paper has been extensively tested with imaging applications that required acquisitions at up to 700 frames per second and LED duty cycles up to approximately 0.001. For other scenarios, the user should take into consideration the following points:

• The software-based timing of LED pulses is sufficiently accurate for typical imaging applications. For applications that rely on high-precision timing, consider employing hardware-based solutions, such as solutions based on pulse width modulation (PWM).
• An advantage of the software-based timings is that the Teensy code can be easily modified to provide exotic flash sequences, e.g., sequences of flashes with varying inter-pulse delays (Fig. 6).

To conclude, we have developed a low-cost stroboscopic illumination system for transmitted light microscopy that can be easily mounted on a condenser of a standard inverted microscope. Although the system bypasses the full standard Koehler illumination, it nonetheless provides sufficiently uniform illumination suitable for many applications. It is capable of delivering flashes with durations down to approximately 500 ns, which are required in high-throughput microfluidic applications such as deformability cytometry or droplet microfluidics. The shortest flash duration is not limited by the Teensy development board and could be improved by using a different design of the illumination unit. The software-based timing of the pulses allows modifications of pulse sequences without in-depth knowledge of the hardware. Due to its low cost, the system can also be used to refurbish old microscopes for which spare parts are no longer available.

## Ethics statements

The work complies with the ethical guidelines of HardwareX and did not involve human subjects nor animal experiments.

## CRediT authorship contribution statement

**Marko Tuljak:** Conceptualization, Investigation. **David Lajevec:** Software. **Špela Zemljič Jokhadar:** Investigation. **Jure Derganc:** Conceptualization, Supervision, Formal analysis, Software. **Rok Štanc:** Conceptualization.

## Declaration of Competing Interest

The authors declare that they have no known competing financial interests or personal relationships that could have appeared to influence the work reported in this paper.
