# Supplementary material for: Low-cost programable stroboscopic illumination with sub-microsecond pulses for high-throughput microfluidic applications
Source: HardwareX. 2022 Oct 4;12:e00367. doi: 10.1016/j.ohx.2022.e00367 (PMC9552099; doi:10.1016/j.ohx.2022.e00367)
Supplement: Supplementary data 1 [file mmc1.pdf]

Logo

Part Number:

Comments:

Date: 25. 05. 2022

Revision:

Designed By:

Material:

Weight:

APPROVALS		
TITLE	NAME	DATE
DRAWN		
CHECKED		
APPROVED		

GENERAL NOTES

UNLESS OTHERWISE SPECIFIED:
DIMENSIONS ARE IN MILLIMETERS
MODEL IS REQUIRED TO COMPLETE PRODUCT DEFINITION FOR ALL NON-DIMENSIONED FEATURES IN THIS DOCUMENT
ALL DIMENSIONS OBTAINED FROM THE MODEL ARE BASIC UNLESS OTHERWISE SPECIFIED
THE GENERAL TOLERANCE FOR ALL UNTOLERANCED SURFACES = 0.2MM
INTERPRET DRAWING IN ACCORDANCE WITH ASME Y14.5-2009, AND ASME Y14.100-2013

Logo

Part Number:

Comments:

Date: 25. 05. 2022

Revision:

Designed By:

Material:

Weight:

APPROVALS		
TITLE	NAME	DATE
DRAWN		
CHECKED		
APPROVED		

GENERAL NOTES

UNLESS OTHERWISE SPECIFIED:
DIMENSIONS ARE IN MILLIMETERS
MODEL IS REQUIRED TO COMPLETE PRODUCT DEFINITION FOR ALL NON-DIMENSIONED FEATURES IN THIS DOCUMENT
ALL DIMENSIONS OBTAINED FROM THE MODEL ARE BASIC UNLESS OTHERWISE SPECIFIED
THE GENERAL TOLERANCE FOR ALL UNTOLERANCED SURFACES = 0.2MM
INTERPRET DRAWING IN ACCORDANCE WITH ASME Y14.5-2009, AND ASME Y14.100-2013
